# Global catastrophic risk from lower magnitude volcanic eruptions

**DOI:** 10.1038/s41467-021-25021-8

**Published:** 2021-08-06

**Authors:** Lara Mani, Asaf Tzachor, Paul Cole

**Affiliations:** 1 Centre for the Study of Existential Risk, University of Cambridge, Cambridge, UK; 2 School of Sustainability, The Interdisciplinary Center (IDC) Herzliya, Herzliya, Israel; 3 School of Geography, Earth and Environmental Science, University of Plymouth, Plymouth, UK

**Keywords:** Natural hazards, Interdisciplinary studies

## Abstract

Globalisation supports the clustering of critical infrastructure systems, sometimes in proximity to lower-magnitude (VEI 3–6) volcanic centres. In this emerging risk landscape, moderate volcanic eruptions might have cascading, catastrophic effects. Risk assessments ought to be considered in this light.

Within the volcanic risk literature, the typical focus of attention for global-scale catastrophes has been on large-scale eruptions with a volcanic explosivity index (VEI) of 7–8^1,2^, which remain relatively rare^3^. The relationship between volcanic eruptions of this scale and global catastrophic risks (GCRs) – events that might inflict damage to human welfare on a global-scale^4^ provided rationality for this tendency. We define this correlation as a ‘VEI-GCR symmetry’, whereby as the magnitude of an eruption increases so too does the probability of a GCR event. The eruption of Tambora in 1815 (VEI 7) is an example of the mechanism that governs the VEI-GCR symmetry, in which a large release of sulfur into the stratosphere brought about periodic global cooling, widespread frosts in the northern hemisphere, and crop failures across Europe^5,6^. This VEI-GCR symmetry has historically defined society’s relationship with volcanoes. Indeed, we have often failed to consider lower-magnitude VEI eruptions as constituting GCRs.

Here, we argue that this symmetry has become imbalanced towards ‘VEI-GCR asymmetry’, driven by clustering of our global critical systems and infrastructures in proximity to active volcanic regions. Critical systems and infrastructures, such as shipping passages, submarine cables, and aerial transportation routes, are essential to sustain our societies and to ensure their continued development^7,8^. We observe that many of these critical infrastructures and networks converge in regions where they could be exposed to moderate-scale volcanic eruptions (VEI 3–6). These regions of intersection, or *pinch points*, present localities where we have prioritised efficiency over resilience, and manufactured a new GCR landscape, presenting a new scenario for global risk propagation.

## A manufactured global catastrophic risk landscape

We saw an example of the VEI-GCR asymmetry mechanism in play during the 2010 VEI 4 eruption of Eyjafjallajökull, Iceland, whereby a moderate-scale volcanic eruption occurred in proximity to a pinch point of critical systems and networks, resulting in global-scale impacts. During the explosive phase of the event, plumes of volcanic ash were transported on north-westerly winds towards continental Europe^9^, resulting in the closure of European airspace, at a loss of US$5 billion to the global economy^10^. This eruption remains the most costly volcanic eruption ever recorded, even when compared to the VEI 6 1991 eruption of Mount Pinatubo, which was the second-largest eruption (in terms of tephra ejected) in the last century. The Mount Pinatubo eruption, by contrast, resulted in economic impacts of around US$374 million (US$740 million in 2021, recalculated for inflation), despite the eruption being 100 times greater in scale. However, increased globalisation and demand for vital commodities that sustain our societies increased the criticality of the trade and transport networks disabled by the Eyjafjallajökull eruption, driving the global economic impacts and demonstrating an imbalance in humanity’s relationship with volcanoes, towards VEI-GCR asymmetry.

**Fig. 1:**
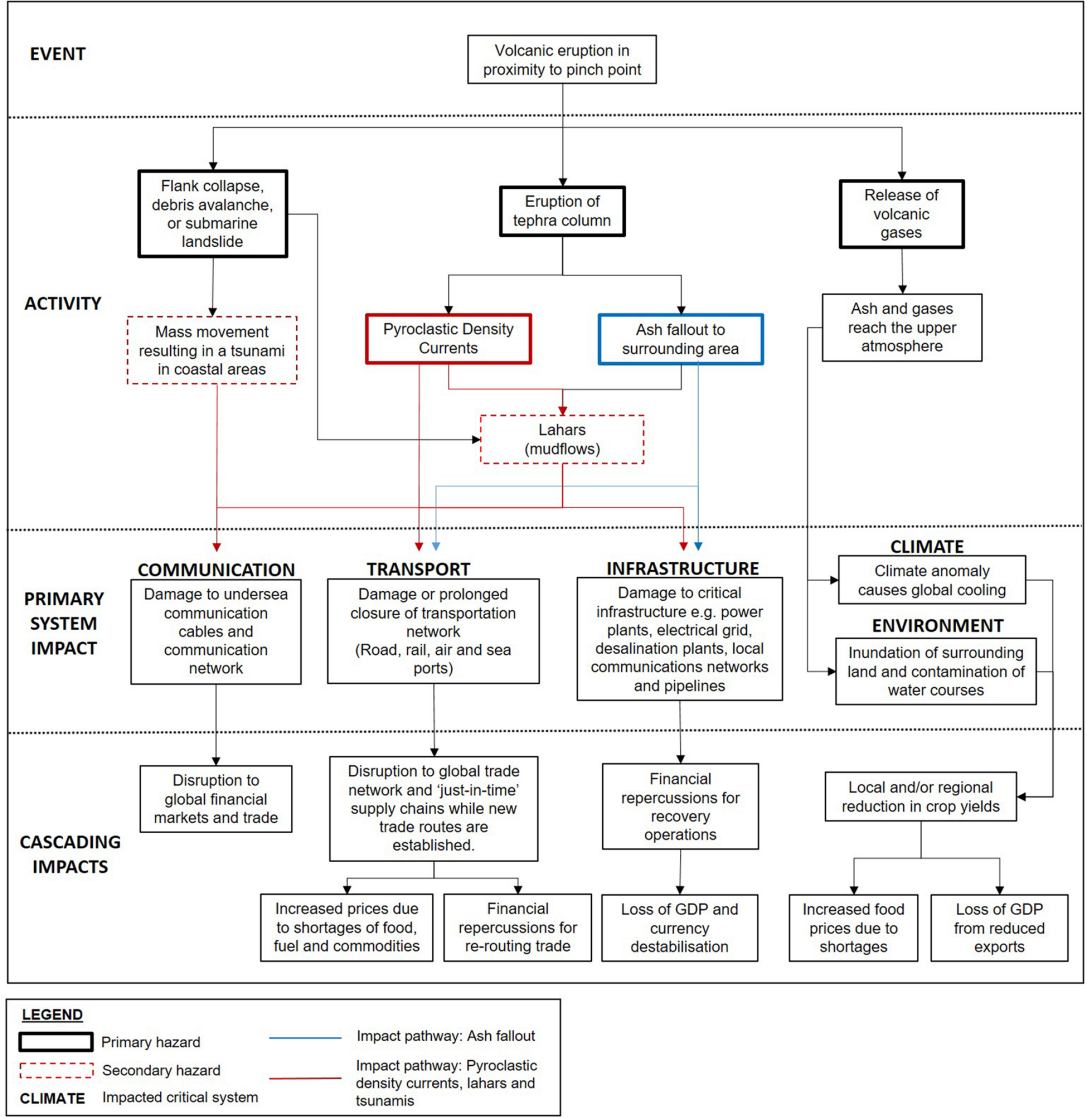
Cascading system failures from lower magnitude volcanic eruptions. Event tree of lower-magnitude (Volcanic Explosivity Index 3–6) volcanic eruptions in proximity to global critical systems. The event tree demonstrates the propagation of cascading failures of related and interlinked critical systems, due to various eruptive hazards, such as the eruption of tephra column, ash fallout, and pyroclastic density currents, as discussed in this Comment. The figure identifies the pathways from systems that are directly vulnerable to such activities to secondary and tertiary knock-on ramifications for interlinked systems. The blue boxes and arrows depict the impact pathway linked to volcanic ash fallout, whilst the red boxes and arrows show the impact pathway for pyroclastic density currents (PDCs), lahars, and tsunamis. Thick black outlined boxes present primary hazards whilst dashed outline boxes identify secondary hazards.

Currently, there is little consideration within existing literature of the interplay between critical systems and lower-magnitude volcanic activity (VEI 3–6) that mark the new GCR geography, with only a few recent studies mentioning this significant link at all^1,3^. Where reference is made, it typically focuses on the larger-scale eruption scenarios (VEI 7 and above), and their direct impacts, such as loss of life and damage to infrastructures^2,5,11^. However, these studies fail to extend the risk assessments further to consider cascading failure mechanisms that can catapult local systems failures to GCR^11^. Figure 1 illustrates potential cascading system failures with global ramifications that could result from moderate volcanic eruptions of VEI of 3 to 6.

## Seven global pinch points

**Fig. 2:**
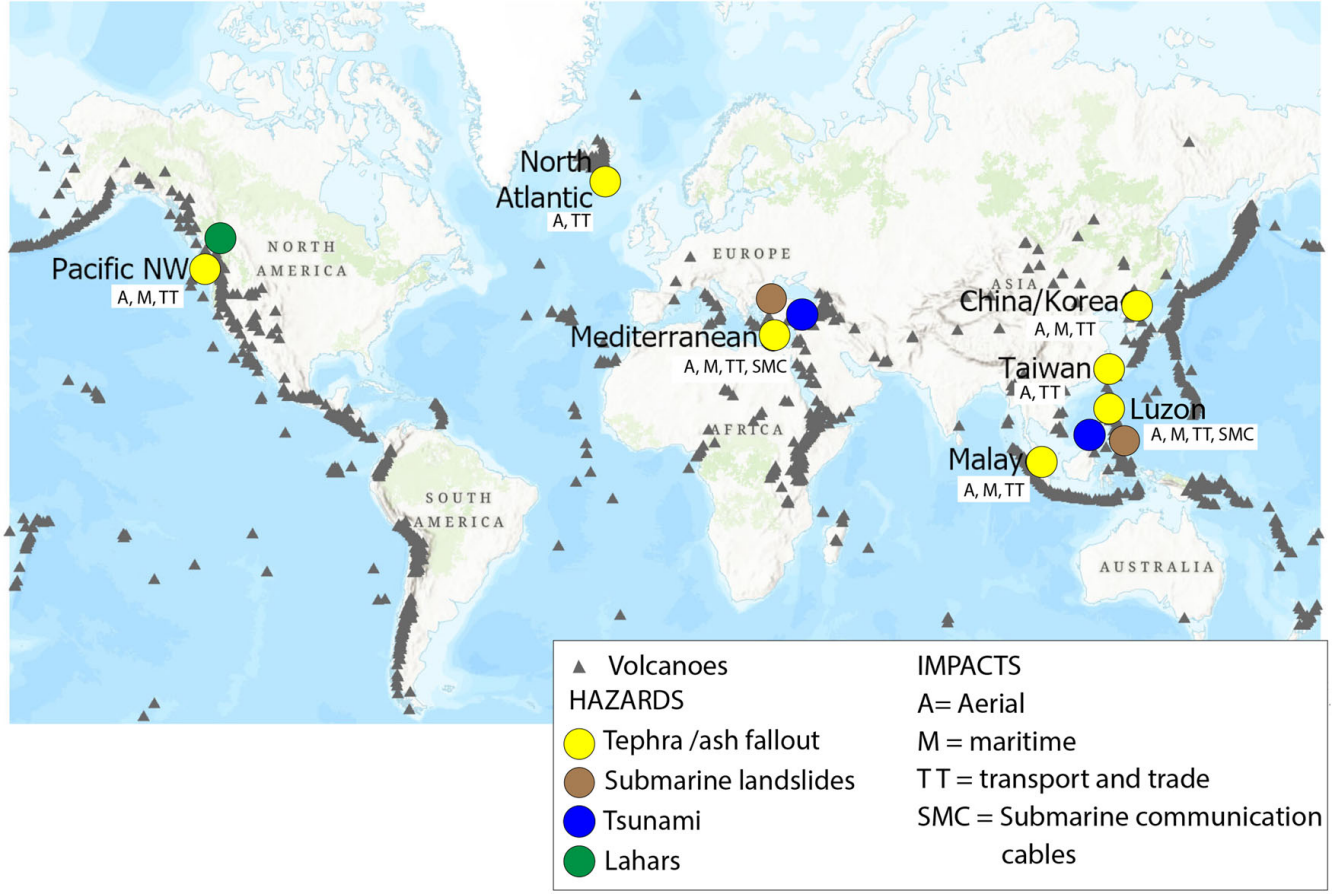
Seven global pinch points. Map of regions or pinch points where clustering of critical systems and infrastructures converge with regions of lower-magnitude volcanic activity (volcanic explosivity index 3–6). These pinch points are presented with the likely associated volcanic hazard activities in circles; where yellow is tephra/ash fallout, brown is submarine landslides, blue are tsunamis, and green are lahars. Each pinch point also includes the potentially impacted systems, including aerial (A), maritime (M), trade and transportation networks (TT), and submarine cables (SMC).

On the consideration of an emerging, asymmetric volcanic risk landscape, we highlight at least seven geographical locations, or pinch points, where a convergence of one or more of critical systems occurs, and delineate the particular GCR mechanism each might provoke. These seven pinch points, shown in Figure 2, identify localities where we perceive the highest levels of criticality for the global systems and infrastructures they encompass, (e.g. shipping passages with high traffic volumes that cannot be easily re-routed).

### Taiwanese pinch point

The Tatun Volcanic Group (TVG) lies on the northern tip of Taiwan and on the edge of metropolitan Taipei. This volcanic complex was historically active between 2.8 and 0.2 Ma; however, new evidence suggests that it has remained active, with frequent episodes of volcanic-tectonic earthquakes^12^. Taiwan is home for the main manufacturing centre of TSMC, the leading producers of over 90% of the most advanced chips and nodes (equivalent to US$18.9 billion market share)^13^ and principal suppliers to the global technology and car industries. An explosive volcanic eruption at TVG could blanket the area in thick tephra deposits, forcing the closure of transportation networks, including the Port of Taipei, essential to TSMC’s supply chain. Prolonged rupture of critical infrastructures such as the electrical grid that supplies the TSMC manufacturing plants could also cause grave disruption to the global supply of chips and nodes, with severe knock-on implications to the global technology industry and global financial markets.

### Chinese–Korean pinch point

The Changbaishan volcanic complex encompassing Mount Paektu straddles the Chinese-North-Korean border, and is most known for its 946 C.E. ‘millennium eruption’ which was estimated to be a VEI 7 eruption. Tephra deposits from this eruption have been documented as far as Hokkaido, Japan^14^, demonstrating the capability of this volcano to cause widespread disruption in the region. An eruption column, even from a smaller-scale eruption (VEI 4–6) at Mount Paektu could be capable of producing a tephra column that would disrupt some of the busiest air routes in the world, such as Seoul to Osaka and Seoul to Tokyo^15^ and to maritime traffic traversing the Sea of Japan.

### Luzon pinch point

The Luzon Strait is a key shipping passage connecting the South China Sea to the Philippine Sea, and a key route for submarine cables, with at least 17 cables connecting China, Hong Kong, Taiwan, Japan, and South Korea. The Luzon Volcanic Arc (LVA) encompassing Mount Mayon, Mount Pinatubo, Babuyan Claro, and Taal volcanoes, among others, presents a possible location for an explosive eruption to disrupt the Strait. Volcanic ash and volcanically-induced submarine landslides and tsunamis in this region (particularly from submarine volcanic centres) would pose a risk to submarine cable infrastructure within the Strait, and result in the closure of the shipping passage. The 2006 7.0 Mw Hengchun earthquake off the south-west coast of Taiwan triggered submarine landslides that severed 9 submarine cables in the Strait of Luzon which connects Hong Kong, China, Taiwan, the Philippines, and Japan, resulting in near-total internet outages and severely disabling communication capacities (up to 80% in Hong Kong), with knock-on widespread disruptions to global financial markets. These disruptions continued for weeks in the aftermath, with repairs to the cables taking 11 ships 49 days to restore^16^.

### Malay pinch point

The Strait of Malacca is one of the busiest shipping passages in the world, with 40% of global trade traversing the narrow route each year^17^. Kuala Lumpur and Singapore both border the Strait and comprise busy aerial and maritime travel and trade hubs. The region is also one of the busiest airspaces in the world, with the aerial route between both cities alone comprising over 5.5 million seats per year^15^. This region is also known to be highly volcanically active, with numerous volcanic centres present along the Indonesian archipelago, such as Mount Sinabung (VEI 4) and Mount Toba in Sumatra, and Mount Merapi (VEI 4) in Central Java. Rupture or either aerial or maritime transportation as a result of a tephra column, could result in severe delays and disruption to global trade. Modelling for a VEI 6 eruption at Mount Merapi which only considered the cost of disruption to aerial routes, with the closure of airspace across Malaysia, Indonesia, and Singapore, estimated a potential loss of up to US$2.51 trillion dollars of global GDP output loss over a 5-year period^18^.

### Mediterranean pinch point

Similar to the Straits of Malacca, The Mediterranean is a vital passage for the maritime transportation of goods and commodities from the Middle East and Asia to Europe, and hosts a large network of submarine communications cables connecting Europe to Africa, North America, the Middle East, and Asia. A volcanically-induced tsunami from a volcanic centre such as Santorini (as happened during the Minoan eruption 3500 BCE), could cause widespread damage to submarine cables and disruption to port facilities and global shipping passages, such as the Suez Canal. The criticality of the Suez Canal was highlighted by the closure of the passage as a result of the stranding of a container ship in March 2021. The 6-day closure is estimated to have cost between US$6–10 billion a week to global trade, through delays in cargo transportation and diversion of ships away from the canal^19^. Numerous volcanic centres in the region are able to produce such activity, including Mount Vesuvius, Santorini, and Campi Flegrei, which are all capable of the explosive eruption of VEI 3–6. Additionally, any tephra column produced during an eruption would result in a provisional closure of European airspace, within widespread delays to aerial transport and trade networks.

### North Atlantic pinch point

The aerial traffic between London and New York comprises over 3 million seats per year^15^. Disruption to this critical artery could cause widespread disruption and delay to global trade and transportation networks. Volcanic centres in Iceland are a potential source for this disruption, with numerous volcanic centres producing explosive events of VEI 3–6, including Katla (1918), Hekla (1947), and Grímsvötn (2011).

### Pacific Northwest pinch point

An eruption of a Cascades volcano, such as Mount Rainier, Glacier Peak, or Mount Baker in Washington, would have the potential to trigger mass flows, such as debris avalanches or lahars, resulting from the melting of glaciers and ice caps, with the potential to reach Seattle^20^. The Osceola mudflow generated around 5600 years ago at Mount Rainier travelled over 60 miles to reach Puget Sound at the site of the present-day Port of Tacoma, Seattle. The generation of a similar-scale mass flow, and combined with any ash fall towards Seattle, would force provisional closure of airports and seaports, which account for 2.5% of the US’s total traffic respectively^18^. Volcanic ash might also affect wider airspace including parts of Canada, including Vancouver, and US cities such as Portland. Scenario modelling for a VEI 6 eruption at Mount Rainier with volcanic ash closing airspace across the northern USA and parts of Canada predict potential losses of up to US$7.63 trillion dollars of global GDP output loss over a 5-year period^18^.

## Reconsidering volcanic risk assessments

By converging critical systems within pinch point localities and placing them at the interface with regions of potential volcanic activity, we have manufactured a new type of GCR from lower VEI 3 to 6 magnitude eruptions; a narrative that has previously been neglected by the volcanic risk community. The identification of ‘pinch points’ tilts the relationship between volcanic activity and GCRs, towards VEI-GCR asymmetry, thereby presenting a current gap in our approach to volcanic risk assessment, and disaster prevention and mitigation practices. We suggest that the community should now consider this risk asymmetry in assessments, and work to fully understand the systemic vulnerabilities that may catapult us from a lower magnitude volcanic eruption (VEI 3 to 6) to a GCR.

As preparedness measures in the pre-disaster phase, we propose that systems mapping and evidence-based foresight activities, such as horizon scanning and event tree analysis be more systematically incorporated into work to identify the full extent and nature of our VEI-GCR asymmetry, and identify opportunities where resilience can be built towards global catastrophic volcanic risk. These activities ought to rely on expert elicitation, including from natural and geophysical sciences, civil engineering, and economics. The asymmetry mechanism discussed here in the context of volcanic hazards is also likely applicable to other geophysical phenomena; a similar approach could be considered for seismic, hydrogeological, and meteorological hazards alike, where this is not already the case.

Unlike super-volcanic eruption scenarios where we have little opportunity for prevention, we can work to reduce the fragility and exposure of our critical systems to rapid-onset natural events, and ultimately increase our resilience to GCRs.
